# High-Level Extracellular Production of a Trisaccharide-Producing Alginate Lyase AlyC7 in *Escherichia coli* and Its Agricultural Application

**DOI:** 10.3390/md22050230

**Published:** 2024-05-18

**Authors:** Xiao-Han Wang, Yu-Qiang Zhang, Xin-Ru Zhang, Xiao-Dong Zhang, Xiao-Meng Sun, Xiao-Fei Wang, Xiao-Hui Sun, Xiao-Yan Song, Yu-Zhong Zhang, Ning Wang, Xiu-Lan Chen, Fei Xu

**Affiliations:** 1 State Key Laboratory of Microbial Technology, Marine Biotechnology Research Center, Shandong University, Qingdao 266237, China; wangxiaohan008002@163.com (X.-H.W.); yqzh1989@sina.com (Y.-Q.Z.); 202232620@mail.sdu.edu.cn (X.-R.Z.); 202312555@mail.sdu.edu.cn (X.-D.Z.); 202212539@mail.sdu.edu.cn (X.-F.W.); sxh19961219@163.com (X.-H.S.); xysong@sdu.edu.cn (X.-Y.S.); zhangyz@sdu.edu.cn (Y.-Z.Z.); 2 Frontiers Science Center for Deep Ocean Multispheres and Earth System, College of Marine Life Sciences, Ocean University of China, Qingdao 266003, China; sunxiaomeng@stu.ouc.edu.cn; 3 Joint Research Center for Marine Microbial Science and Technology, Shandong University and Ocean University of China, Qingdao 266237, China; 4 Shandong Key Laboratory of Marine Ecological Restoration, Shandong Marine Resource and Environment Research Institute, Yantai 264006, China

**Keywords:** alginate lyase, alginate oligosaccharide, alginate trisaccharide, extracellular production, root growth

## Abstract

Alginate oligosaccharides (AOS), products of alginate degradation by endotype alginate lyases, possess favorable biological activities and have broad applications. Although many have been reported, alginate lyases with homogeneous AOS products and secretory production by an engineered host are scarce. Herein, the alginate lyase AlyC7 from *Vibrio* sp. C42 was characterized as a trisaccharide-producing lyase exhibiting high activity and broad substrate specificity. With PelB as the signal peptide and 500 mM glycine as the additive, the extracellular production of AlyC7 in *Escherichia coli* reached 1122.8 U/mL after 27 h cultivation in Luria-Bertani medium. The yield of trisaccharides from sodium alginate degradation by the produced AlyC7 reached 758.6 mg/g, with a purity of 85.1%. The prepared AOS at 20 μg/mL increased the root length of lettuce, tomato, wheat, and maize by 27.5%, 25.7%, 9.7%, and 11.1%, respectively. This study establishes a robust foundation for the industrial and agricultural applications of AlyC7.

## 1. Introduction

Alginate, a natural polysaccharide, is abundantly present in the cell walls of marine brown algae, such as *Ectocarpus*, *Laminaria*, and *Sargassum* [1,2,3]. Alginate is composed of β-D-mannuronate (M) and α-L-guluronate (G) residues connected by 1,4 glycosidic bonds, resulting in three different blocks: poly-mannuronate (PM) blocks, poly-guluronate (PG) blocks, and poly-mannuronate and guluronate (PMG) blocks [4,5]. The structural characteristics of alginate, including the sequence of monomers, M to G ratio, block length, and molecular weight, exhibit significant variations attributed to their source and growth conditions [6,7,8]. Alginate has widespread application due to its biocompatibility, hypotoxicity, cost-effectiveness, and capacity to gel when exposed to divalent cations [9,10]. Nonetheless, the high molecular weight, high viscosity, and complex structure of alginate impose limitations on its performance, especially in biomedical, functional food, and agricultural fields [11].

Alginate oligosaccharides (AOS) are products derived from the degradation of alginate. Numerous studies have shown that AOS, with their low molecular weights, possess highly favorable biological activities [12,13,14]. The variations in AOS composition contribute to their diverse activities, rendering them versatile for a wide range of applications. Notably, as a natural substance derivative without observable toxicity, AOS show significant promise for agricultural applications. They can boost plant growth, reduce growth suppression from environmental stress, trigger plant defense mechanisms, and prolong fruit shelf life [12,15,16].

Current AOS preparation methods primarily involve physical, chemical, and enzymatic methods [17]. Among these, enzymatic methods utilizing endotype alginate lyase offer advantages such as safety, environmental friendliness, and improved preservation of active groups in the alginate substrate [17,18]. Alginate lyase, which is a type of polysaccharide lyase (PL), cleaves glycosidic bonds between alginate monomers based on their substrate preferences, generating unsaturated double bonds at the non-reducing end. The substrate preferences of alginate lyases include PM preference, PG preference, and bifunctionality, enabling the degradation of various glycosidic bonds within alginate. However, despite the merits of the enzymatic method for AOS production, two challenging issues impede its development. Firstly, the products of endotype alginate lyases are typically a mixture of AOS at degrees of polymerization (DPs) ranging from 2 to 6 [19,20,21]. The considerable heterogeneity of AOS products results in instability and variability regarding their biological activity. Secondly, while numerous alginate lyases have been overexpressed in the cytoplasm of the engineered strains [22,23], e.g., *Escherichia coli*, secretory production of recombinant proteins is highly regarded in industry due to its advantages such as simplified downstream processing at a low cost, efficient productivity, high activity levels, and stability [22]. Nevertheless, targeting proteins in the extracellular space has been constrained by complex secretory mechanisms and the limited intrinsic secretory capacities of strains. Therefore, the exploitation of alginate lyases with homogeneous products and secretory production is of great significance for the industrial and agricultural applications of alginate lyases.

The aim of this study was to develop an alginate lyase that can efficiently produce homogeneous AOS and can be extracellularly produced by an engineered host. In this study, the alginate lyase AlyC7 from the marine bacterium *Vibrio* sp. C42, an alginate-degrading strain isolated from a *Sargassum* sample [24], was identified as a bifunctional alginate lyase exhibiting specificity in generating trisaccharides as the main product. A secretory expression system of AlyC7 by *E. coli* was then constructed, and the expression conditions were optimized. Using the AlyC7 secreted by recombinant *E. coli*, a process to produce AOS with trisaccharides as the main component was set up based on optimization of the enzymolysis conditions. Further analysis showed that the prepared AOS had a significant promotional effect on the root growth of several plant seeds. The results indicate that the alginate lyase AlyC7 has a promising potential in the production of alginate trisaccharides, laying a solid foundation for its industrial and agricultural applications.

## 2. Results and Discussion

### 2.1. Sequence Analysis and Biochemical Characterization of AlyC7

**Figure 1:**
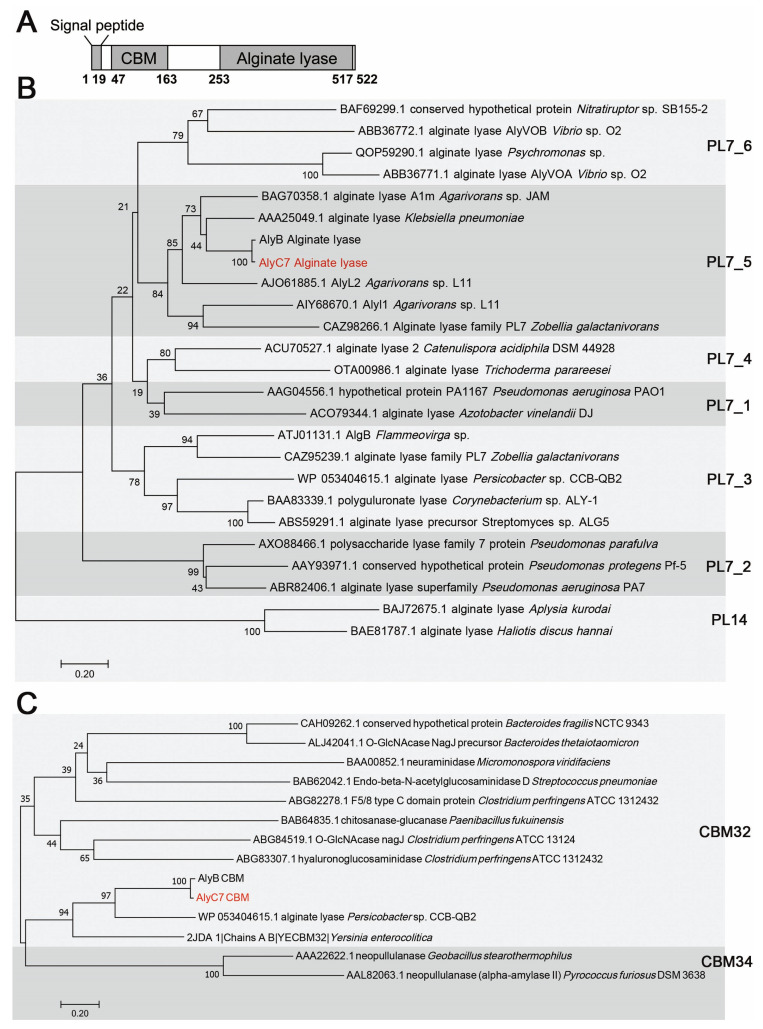
Sequence analysis of the alginate lyase AlyC7. (**A**) Schematic domain diagram of AlyC7. (**B**,**C**), phylogenetic analysis of the catalytic domain (**B**) and carbohydrate-binding module (**C**) of AlyC7. The phylogenetic trees were generated using MEGA 7 software through the neighbor-joining approach. Bootstrap analysis with 1000 replicates was performed.

The gene *alyC7* from *Vibrio* sp. C42 is 1569 bp in length and encodes a putative alginate lyase, AlyC7, comprising 522 amino acid residues, including a predicted 19-residue signal peptide (Figure 1A). Blast analysis against the NCBI conserved domain database revealed that AlyC7 contained two domains, a carbohydrate-binding module (CBM, Thr47-Leu163) and an alginate lyase domain (Phe253-His517), which were phylogenetically classified within the CBM32 family and subfamily 5 of the PL7 family, respectively (Figure 1B,C), suggesting that AlyC7 is a PL7 alginate lyase. AlyC7 shares the highest sequence similarity of 94.14% with AlyB, a PL7 alginate lyase also composed of the CBM32 and alginate lyase domains [25].

**Figure 2:**
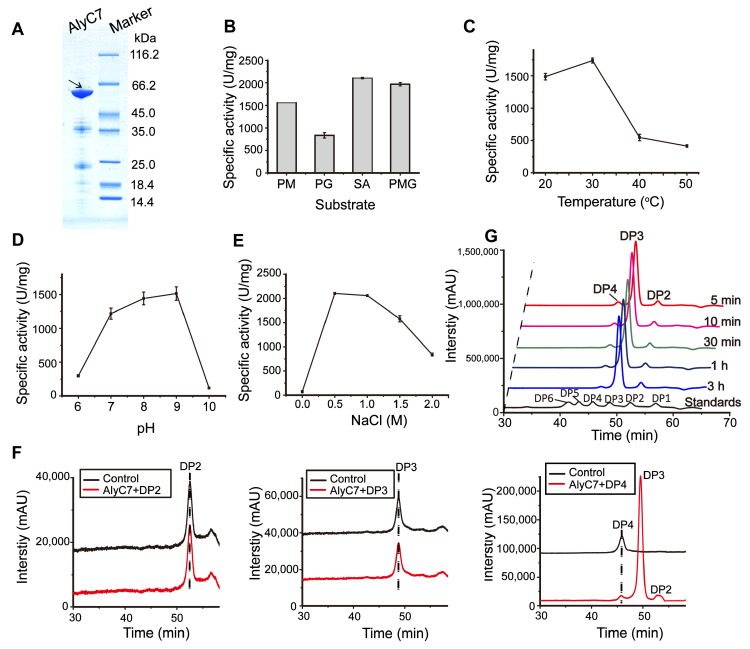
Heterologous expression and biochemical characterization of the alginate lyase AlyC7. (**A**) SDS-PAGE analysis of the purified recombinant alginate lyase AlyC7. The arrow indicates the AlyC7 band. (**B**) Substrate specificity of AlyC7. SA, sodium alginate. (**C**) The effect of temperature on AlyC7 activity. (**D**) The effect of pH on AlyC7 activity. (**E**) The effect of NaCl concentration on AlyC7 activity. The data shown in the graphs (**C**–**E**) are from triplicate experiments (mean ± standard deviation [S.D.]). (**F**) Analysis of the minimal substrate of AlyC7. Commercial saturated mannuronate oligosaccharides were used as a control. (**G**) Time-course degradation of AlyC7 towards sodium alginate. DP, degree of polymerization. DP1 to DP6 represent mono-, di-, tri-, tetra-, penta- and hexa-saccharide, respectively. Figures in (**F**,**G**) are representatives of triplicate experiments.

To characterize AlyC7, *alyC7* excluding the signal peptide sequence was intracellularly expressed in *E. coli* BL21 (DE3), and the recombinant AlyC7 was purified. The resultant AlyC7 exhibited an apparent molecular mass of 55 kDa based on SDS-PAGE analysis, consistent with its theoretical molecular mass of 55.21 kDa (Figure 2A). AlyC7 degraded various alginate substrates, including PM, PG, sodium alginate, and PMG, with a preference for sodium alginate, suggesting that it is a bifunctional alginate lyase (Figure 2B). The highest activity of AlyC7 was observed at 30 °C, with more than 80% activity retention at 20 °C and a substantial decline at 40 °C (losing over 70% of its maximal activity) (Figure 2C). In addition, AlyC7 showed the highest activity at pH 9.0 and retained at least 80% of its activity between pH 7.0 and 9.0 (Figure 2D). Moreover, the activity of AlyC7 was significantly enhanced by NaCl, peaking at a concentration of 0.5 M and showing a 28-fold increase compared to that in the absence of NaCl (Figure 2E). These results suggested AlyC7’s adaptation to the alkaline and saline marine environment, which *Vibrio* sp. C42 was from [24]. Under the optimal reaction conditions, the maximum activity of AlyC7 reached 2104.57 U/mg.

AlyC7 could break down tetrasaccharides into smaller products but not trisaccharides (Figure 2F), suggesting that the minimal substrate for AlyC7 is a tetrasaccharide. Time-course degradation assays with sodium alginate revealed the specificity of AlyC7 for trisaccharide production. After 1 h of degradation, trisaccharides accounted for more than 80% of the degradation products, as determined by analyzing the peak area in the gel filtration chromatogram (Figure 2G). While most characterized alginate lyases yield a mixture of oligosaccharides ranging from DP2 to DP6, only a few predominantly produce trisaccharides [1,26,27]. For instance, AlyF from *Vibrio splendidus* OU02 and AkAly28 from *Aplysia kurodai*, which were active towards PG and PM, respectively, primarily generated trisaccharides [28,29]. Alg7A from *Vibrio* sp. W13, a bifunctional lyase with the highest activity around 650 U/mg, mainly produced trisaccharides after a 24 h reaction with sodium alginate [30]. AlyM2 from *Pseudoalteromonas* sp. M9 degraded various alginate substrates with an enzyme activity of 11.7–29.7 U/mg, resulting in trisaccharides as the principal products, accounting for nearly 80% of the total [31]. In contrast, AlyC7 exhibits broader substrate specificity than AlyF and AkAly28 and higher enzymatic activity than Alg7A and AlyM2, suggesting the promising potential of AlyC7 for industrial trisaccharide production.

### 2.2. Secretory Expression of AlyC7 in E. coli with Different Signal Peptides

The extracellular production of proteins offers several benefits, particularly in the simplification of purification processes [32,33]. Signal peptide-assisted recombinant protein secretion is a commonly employed technique for extracellular protein production [32,34,35]. In Gram-negative bacteria, proteins can be secreted across the cell envelope mainly via the types I–VI secretion pathways, with the type II pathway being the most prevalent in *E. coli* [33,36]. To explore the secretion efficiency of AlyC7 in *E. coli*, five signal peptides associated with the type II pathway (PelB, MalE, OmpA, PhoA, and OmpT), as well as AlyC7’s native signal peptide, were evaluated. Plasmids harboring *alyC7* with these signal peptide sequences were constructed and transformed into *E. coli* BL21 (DE3), resulting in recombinant strains designated as PelB-AlyC7, MalE-AlyC7, OmpA-AlyC7, PhoA-AlyC7, OmpT-AlyC7, and WT-AlyC7. The secretion of the recombinant proteins was then evaluated by SDS-PAGE analysis and enzymatic activity measurement.

**Figure 3:**
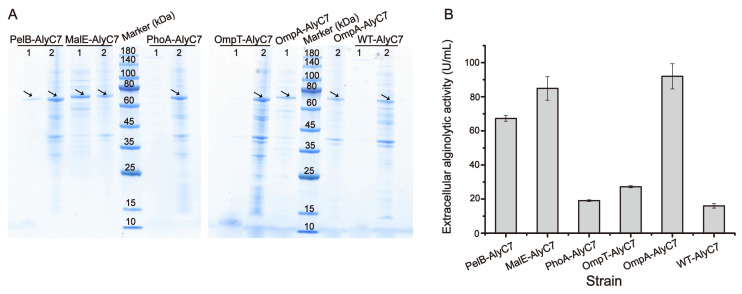
Secretory expression of AlyC7 mediated by different signal peptides in *E. coli*. (**A**) SDS-PAGE analysis of the expression and secretion of AlyC7 with different signal peptides in the recombinant *E. coli* strains. Lane 1, extracellular fraction; lane 2, intracellular fraction. The arrows indicate the AlyC7 bands. (**B**) Extracellular alginolytic activity of the recombinant *E. coli* strains harboring AlyC7 with different signal peptides. The data shown in the graph are from triplicate experiments (mean ± standard deviation [S.D.]).

All six recombinant proteins were successfully expressed in the recombinant BL21 (DE3) strain based on SDS-PAGE analysis (Figure 3A). However, only extracellular samples (fermentation broth supernatants) from strains PelB-AlyC7, MalE-AlyC7, and OmpA-AlyC7 displayed a clear AlyC7 band on the SDS-PAGE gels (Figure 3A). Correspondingly, these samples showed significantly higher alginolytic activity than those from the other three strains (Figure 3B). These results indicated that the signal peptides PelB, MalE, and OmpA obviously facilitated the extracellular secretion of AlyC7 from *E. coli*.

### 2.3. Effects of Additives on AlyC7 Secretion by E. coli

**Figure 4:**
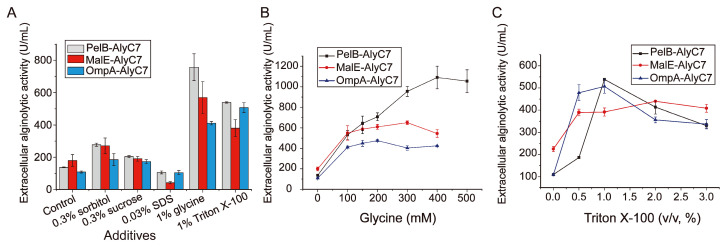
Effects of additives on AlyC7 secretion by three recombinant *E. coli* strains. (**A**) Effects of different additives on AlyC7 secretion. Cultures without the addition of any additives were used as controls. (**B**) Effects of glycine concentrations on AlyC7 secretion. (**C**) Effects of Triton X-100 concentrations on AlyC7 secretion. The effects of the additives on AlyC7 secretion in (**A**–**C**) were analyzed by measuring the extracellular alginolytic activities of the culture supernatants by the DNS method. The data shown in the graphs are from triplicate experiments (mean ± standard deviation [S.D.]).

Supplementation of certain chemicals, such as amino acids or surfactants, in the *E. coli* culture medium has been documented to enhance protein secretion by modulating periplasmic osmolality and altering the integrity of the cell wall [37,38,39]. To promote the secretion of AlyC7, different additives, including glycine, Triton X-100, sorbitol, sucrose, and SDS, were added to the cultures of the strains PelB-AlyC7, MalE-AlyC7, and OmpA-AlyC7, and their effects on AlyC7 secretion were assessed by quantifying the extracellular alginolytic activity of the cultures. The presence of glycine or Triton X-100 in the culture media significantly elevated the extracellular alginolytic activities of all the recombinant strains compared to sorbitol, sucrose, or SDS, thus confirming the efficacy of glycine and Triton X-100 in promoting AlyC7 secretion (Figure 4A). Afterwards, the effects of glycine or Triton X-100 concentrations on AlyC7 secretion by the strains were evaluated. With the increase in glycine concentration, the extracellular alginolytic activity of the strains initially rose and then plateaued, with PelB-AlyC7 achieving the highest activity (1090 U/mL) at 400 mM glycine (Figure 4B). Differently, with the increase in Triton X-100 concentration, the extracellular alginolytic activity of the strains increased, peaked, and then diminished, implying that high concentrations of Triton X-100 might negatively affect *E. coli* cell growth (Figure 4C). Notably, strain PelB-AlyC7 also displayed the highest extracellular alginolytic activity (539 U/mL) with the addition of 1% Triton X-100 (Figure 4C), which, however, was less effective than 400 mM glycine. Hence, strain PelB-AlyC7 was selected for further optimization.

### 2.4. Optimization of Culture Conditions for the Extracellular Production of AlyC7 in Strain PelB-AlyC7

**Figure 5:**
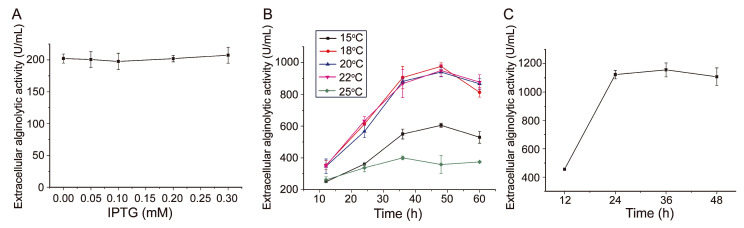
Effects of culture conditions on the extracellular production of AlyC7 in strain PelB-AlyC7. (**A**) effect of the IPTG concentration. (**B**) Effects of induction time and temperature. (**C**) The extracellular production of AlyC7 by strain PelB-AlyC7 cultured in the medium containing 500 mM glycine and induced at 20 °C. The effects of culture conditions on the extracellular production of AlyC7 in (**A**–**C**) were analyzed by measuring the extracellular alginolytic activities of the culture supernatants by the DNS method. The data shown in the graphs are from triplicate experiments (mean ± standard deviation [S.D.]).

It has been shown that culture conditions and inducer concentration may influence extracellular protein production in *E. coli* [34]. To further enhance the extracellular production of AlyC7 in strain PelB-AlyC7, the effects of IPTG concentration, induction temperature (the culture temperature after OD_600_ of the strain reached 0.6), and induction time (the culture time after OD_600_ of the strain reached 0.6) were first investigated by single-factor experiments. The results revealed that IPTG concentration had no significant effect on the extracellular alginolytic activity, likely because trace lactose in LB medium’s peptone component sufficed to induce AlyC7 expression (Figure 5A). Thus, IPTG was unnecessary for AlyC7 expression. However, both induction temperature and time had a significant effect on AlyC7 production, with optimal values being 18–22 °C and 48 h, respectively (Figure 5B). Subsequently, an orthogonal experiment, commonly employed in fermentation process optimization in industrial settings, was conducted to fine-tune the following three influencing factors: glycine concentration, induction temperature, and induction time (Table S1). Based on the detection of the extracellular activity, the optimal conditions for the extracellular production of AlyC7 were 24 h for induction time, 20 °C for induction temperature, and 500 mM for glycine concentration (Table S2). Considering that it took approximately 3 h for the strain culture to reach an OD_600_ of 0.6 since inoculation, the total time for the extracellular production of AlyC7 in strain PelB-AlyC7 was approximately 27 h. As shown in Figure 5C, when strain PelB-AlyC7 was cultured under optimal conditions, the extracellular alginolytic activity reached 1122.8 U/mL (Figure 5C).

To our knowledge, only two studies have documented the secretory expression of alginate lyases to date. Li et al. described the recombinant production of rSAGL, an alginate lyase from *Flavobacterium* sp. H63, in *Pichia pastoris*. The *P. pastoris* strain was cultured in BMGY medium containing 4 × 10^−5^% (*w*/*v*) biotin, 1.34% (*w*/*v*) yeast nitrogen base, 100 mM potassium phosphate (pH 6.0), and 10 g/L glycerol. During fermentation, PTM1 trace salts, glycerol, and methanol were fed in batches. After 168 h, the alginolytic activity of the yeast culture’s supernatant reached a maximum of 915.5 U/mL [23]. In comparison, the extracellular alginolytic activity of strain PelB-AlyC7 was higher, and the fermentation duration was significantly reduced. Unlike the fed-batch method required for *P. pastoris*, PelB-AlyC7 only necessitates glycine supplementation during fermentation. In another study, Meng et al. reported the secretory expression of the alginate lyase Aly01 in *E. coli*, which shares 71.4% sequence similarity with AlyC7 and primarily yields trisaccharides along with small amounts of disaccharides and tetrasaccharides [22]. Aly01’s extracellular production was carried out in TB medium supplemented with 1 mM IPTG, 120 mM glycine, and 10 mM CaCl_2_ at 18 °C for 48 h, achieving an extracellular activity of 796.3 U/mL after unit conversion [22]. Compared with the *E. coli* strain constructed by Meng et al., strain PelB-AlyC7 also exhibited higher extracellular activity. Furthermore, the culture duration was nearly halved without the need for IPTG or metal ions. Overall, PelB-AlyC7 shows several industrial advantages: high extracellular alginolytic activity, short fermentation time, a simplified medium, and a straightforward process, potentially leading to reduced production costs.

### 2.5. AOS Preparation with the Produced AlyC7

**Figure 6:**
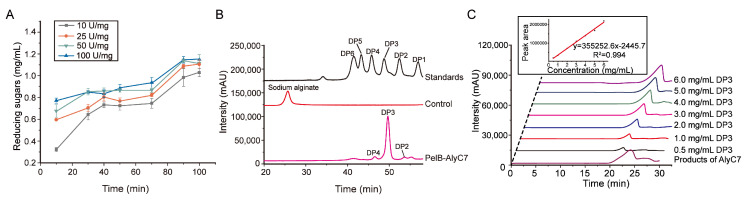
Optimization of enzymatic hydrolysis conditions and analysis of the prepared AOS. (**A**) Effects of the E/S ratio and enzymolysis time on the AOS production. The effects were analyzed by measuring the content of reducing sugars released in the mixture using the DNS method. The graph shows data from triplicate experiments (mean ± standard deviation [S.D.]). (**B**) HPLC analysis of the degradation products of sodium alginate by AlyC7. The degradation reaction was performed at 30 °C for 90 min in a 200 µL mixture containing 0.5 M NaCl, 50 mM Tris-HCL (pH 9.0), 2 mg/mL sodium alginate, and AlyC7 with an E/S ratio of 50 U/mg. DP1 to DP6 represent mono-, di-, tri-, tetra-, penta-, and hexa-saccharide, respectively. Saturated mannuronate oligosaccharides were taken as the standards. (**C**) Analysis of the yield of trisaccharides in the degradation products. The inset is a standard curve correlating trisaccharide concentration to peak area, which was generated using commercial saturated mannuronate trisaccharide (DP3) at concentrations of 0.5–6 mg/mL as standards. Figures in (**B**,**C**) are representatives of triplicate experiments.

The produced AlyC7, namely the extracellular supernatant of strain PelB-AlyC7 cultured under the aforementioned optimized conditions, was then used to prepare AOS from sodium alginate. Sodium alginate was hydrolyzed with AlyC7 at its optimum temperature and pH determined above (Figure 2), and two other enzymatic hydrolysis parameters, the enzyme-substrate ratio (E/S ratio) and enzymolysis time, were further optimized. The optimal E/S ratio was determined to be 50 U/mg, and the ideal enzymolysis time was set at 90 min (Figure 6A). Then, sodium alginate was hydrolyzed by the produced AlyC7 under optimized enzymolysis conditions, and the AOS product underwent HPLC analysis. The result showed that the product contained predominantly trisaccharides (89.56%), with only small amounts of disaccharides (4.32%) and tetrasaccharides (6.12%) (Figure 6B). The yield of trisaccharides was quantified as 758.60 mg/g based on the prepared standard curve (Figure 6C), and the proportion of trisaccharides in the AOS product, referred to as trisaccharide purity, was calculated to be 85.12%.

There have been many studies on the production of AOS by alginate lyases, with degradation times typically spanning 6 to 48 h [23,30,40]. However, the yield of AOS has not been extensively quantified. Jiang et al. achieved AOS production with DP1-DP5 from sodium alginate using the PL17 alginate lyase AlgL17 from *Microbulbifer* sp. alw1. The reaction commenced with sodium alginate at a concentration of 5 mg/mL and an E/S ratio of 6 U/mL for 8 h, followed by an addition of 3 U/mL enzyme for another 9 h. Despite the economical amount of AlgL17, the total enzymolysis duration was lengthy, totaling 17 h [41]. Additionally, the PL6 alginate lyase AlyM2 from *Pseudoalteromonas* sp. M9 predominantly broke down sodium alginate into trisaccharides with a yield of 588.4 mg/g after a 6 h reaction [31]. In contrast, AlyC7 requires only 90 min for sodium alginate enzymolysis, yielding more trisaccharides than AlyM2. Thus, AOS production via the produced AlyC7 is characterized by a rapid hydrolysis rate and exceptional homogeneity and purity of the AOS.

### 2.6. Effects of the Prepared AOS on the Root Growth of Plant Seeds

**Figure 7:**
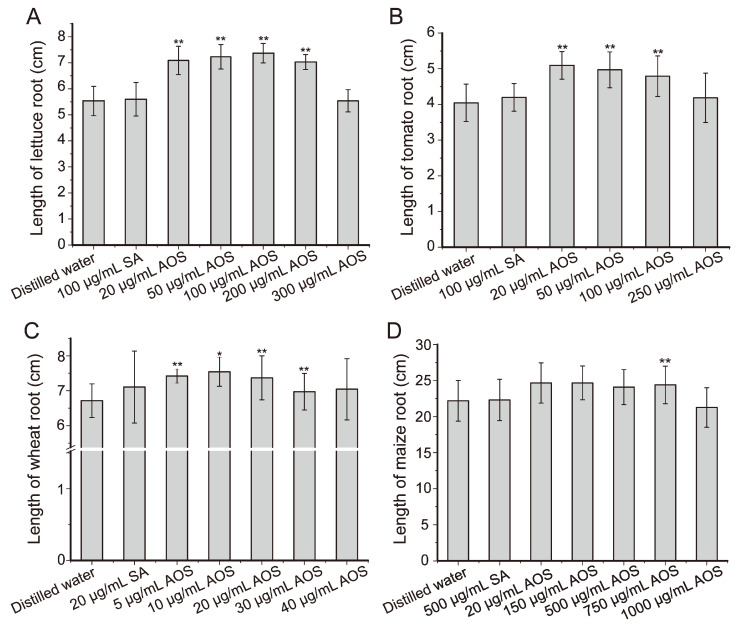
Effects of the prepared AOS on the root length of plant seeds. (**A**) Effects of AOS on the root length of lettuce. Root length was determined on day 7. Each treatment was replicated three times, and 100 seeds were used in each replicate. (**B**) Effects of AOS on the root length of tomato. Root growth was observed on day 5. Each treatment was replicated three times, and 30 seeds were used in each replicate. (**C**) Effects of AOS on the root length of wheat. Root growth was observed on day 4. Each treatment was replicated three times, and 100 seeds were used in each replicate. (**D**) Effects of AOS on the root length of maize. Root growth was observed on day 7. Each treatment was replicated three times, and 25 seeds were used in each replicate. SA, sodium alginate. A paired *t*-test was used to compare the difference between the control groups and the experimental groups. **, significant difference from the control group (distilled water) at *p* < 0.01. *, significant difference from the control group (distilled water) at *p* < 0.05. The data shown in the graphs are from triplicate experiments (mean ± standard deviation [S.D.]).

The beneficial effects of AOS on plants have been extensively reported [12,42]. Nevertheless, diverse preparation methods result in AOS with distinct structural properties, thereby influencing their biological activity [13,43]. In this study, we investigated the effect of the AOS produced by AlyC7 from sodium alginate on the root growth of different plants, including dicots, lettuce and tomato, and monocots, wheat and maize. As shown in Figure 7, compared to the control groups treated with distilled water, the root lengths of the seeds in the experimental groups treated with the AOS exhibited varying degrees of increase, but the seeds treated with sodium alginate did not display a significant increase in root length (Figure 7). Among the seeds, the AOS exhibited the greatest promotional effect on the root growth of lettuce seeds, with increases of more than 27% (≥7.03 cm) in root length at AOS concentrations of 20–200 μg/mL compared to the control (5.53 cm) (Figure 7A). Similarly, the root length of tomato seedlings increased by over 18% (≥4.79 cm) at AOS concentrations of 20–100 μg/mL compared to the control (4.05 cm) (Figure 7B). In comparison, the promotional effect of the AOS on the root growth of monocot seeds, wheat and maize, was less. The AOS at concentrations of 5–30 μg/mL slightly enhanced the root growth of wheat seeds, with the 10 μg/mL treatment being the most effective, resulting in a 12% increase (7.54 cm) compared to the group treated with distilled water (6.72 cm) (Figure 7C). The root length of maize seeds exhibited an approximate 9% increase at AOS concentrations ranging from 20–750 μg/mL compared to the group treated with distilled water (22.19 cm) (Figure 7D). These results demonstrated that the AOS prepared with AlyC7 had a promotional effect on the root growth of plant seeds.

Root systems are crucial for plants, as they provide the necessary area to absorb water and nutrients and serve to anchor the plants in the soil. The promotional effects of AOS on root growth have been studied on several plants. For instance, alginate lyase-lysate (A.L.L.), prepared by degrading sodium alginate with alginate lyases of *Alteromonas macleodii*, was reported to promote the elongation of barley roots, with the effective concentration being 100–3000 μg/mL. The relative root length increased with increasing concentrations of A.L.L. up to 800 μg/mL, and then remained constant (180%) up to 3000 μg/mL [44]. Le et al. produced AOS from alginate by radiation and demonstrated that 100 μg/mL of the prepared AOS enhanced root length by 9.7–39.4% of lisianthus, Limonium, and chrysanthemum [45]. Xu et al. prepared AOS by digesting PG and PM with an alginate lyase from *Pseudoalteromonas* sp. No. 272, generating guluronate oligosaccharides (GOS) and mannuronate oligosaccharides (MOS). The root growth-promoting activity of GOS and MOS on carrot and rice roots was further analyzed. It was observed that only GOS had promoting activity on the roots of both plants, with the maximum activity at 0.75 mg/mL [46]. In addition, Iwasaki et al. used an alginate lyase from *Corynebacterium* sp. to produce AOS with DP2-DP8 and found that 200–3000 μg/mL of AOS doubled lettuce root growth compared to the controls [47]. It can be speculated that the effect of AOS on root elongation varies depending on AOS types and the specific plant species. Some AOS may exhibit a more pronounced stimulative effect on the root growth of certain plants, albeit functioning at high concentrations up to milligrams per milliliter. On the other hand, some AOS can effectively promote root growth at micrograms per milliliter levels, albeit potentially less significantly compared to the aforementioned scenario. AOS prepared by AlyC7 may align with the latter scenario. Therefore, in comparison to AOS produced by Iwasaki et al., AOS produced by AlyC7 displayed a less pronounced effect on lettuce root growth, yet the required AOS quantity was over tenfold lower. However, when comparing the AOS prepared in this study with that derived from irradiated alginate [45], it showed a comparable effect on root growth at even lower concentrations. AOS at a concentration of 20 μg/mL increased the root lengths of lettuce, tomato, wheat, and maize by 27.5%, 25.7%, 9.7%, and 11.1%, respectively (Figure 7). Hence, the AOS produced by AlyC7 may have promising potential in agriculture, as it is not only effective but also used economically.

## 3. Materials and Methods

### 3.1. Materials and Strains

Sodium alginate was purchased from Sigma (Saint Louis, MO, USA). PM and PG were obtained from Zzstandard (Qingdao, China), while oligosaccharide standards were acquired from BZ Oligo Biotech Co., Ltd. (Qingdao, China). PMG was prepared following the previously described method [48]. *Vibrio* sp. C42, isolated from a *Sargassum* sample collected from coastal seawater in Shandong Province, China [24], was preserved in our laboratory, and its genome was deposited in NCBI under the accession number of JAEKGD000000000.1. *E. coli* strains were purchased from Tsingke (Qingdao, China). Seeds of wheat (*Triticum aestivum* L.), maize (*Zea mays* L.), lettuce (*Lacuta sativa*), and tomato (*Lyopersicon esculentum* Mill.) were purchased from the seed wholesale market Jimo, Qingdao, China (36°37′ N, 120°44′ E).

### 3.2. Bioinformatics Analysis

The genomic DNA of *Vibrio* sp. C42 underwent shotgun sequencing on the Illumina Hiseq sequencing platform (Majorbio, Shanghai, China). SignalP 5.0 was employed to predict the signal peptide sequence of AlyC7 [49]. The conserved domains within the alginate lyase AlyC7 were identified via blast searches in the Conserved Domain Database [50]. Phylogenetic trees were constructed with MEGA 7.0 software (version 7.0, Mega Limited, Auckland, New Zealand), using sequences of related alginate lyases, or CBMs.

### 3.3. Plasmid Construction

The genomic DNA of *Vibrio* sp. C42 was extracted using the BioTekeDNA extraction kit (Beijing, China). For the intracellular expression of AlyC7 in *E. coli*, the *alyC7* gene (GenBank: WP_010438370.1) without the signal peptide sequence was PCR-amplified from the genomic DNA of strain C42 and cloned into the pET-22b vector at the *Nde* I and *Xho* I restriction sites, incorporating a C-terminal 6×His-tag. For secretory expression, *alyC7,* along with a signal peptide (AlyC7’s native signal peptide, PelB, MalE, OmpA, PhoA, or OmpT), was similarly cloned into pET-22b for expression with a C-terminal His-tag using the primer pairs listed in Table S3.

### 3.4. Protein Expression and Purification

The engineered plasmids were introduced into *E. coli* BL21 (DE3) for protein expression. Recombinant *E. coli* BL21 (DE3) cells were grown in Luria-Bertani (LB) medium. Intracellular expression of AlyC7 was induced with 0.3 M isopropyl β-D-1-thiogalactopyranoside (IPTG) at 18 °C for 16 h. *E. coli* cells were harvested and disrupted using a JN-02C French press (JNBIO, Guangzhou, China) in a buffer containing 100 mM NaCl and 50 mM Tris-HCl (pH 8.0). After centrifugation at 15,000× *g* at 4 °C for 50 min, the recombinant AlyC7 was first purified via Ni-affinity chromatography (Qiagen, Dusseldorf, Germany) and subsequently subjected to gel filtration chromatography on a Superdex 200 column (GE Healthcare, Chicago, IL, USA) in the buffer comprising 10 mM Tris-HCl (pH 8.0) and 100 mM NaCl. The protein concentration was quantified by using the bicinchoninic acid (BCA) protein assay kit (Thermo, Waltham, MA, USA) with bovine serum albumin as the standard.

### 3.5. Enzymatic Activity Assay

The recombinant AlyC7 activity was assessed via the ultraviolet absorption spectroscopy method [51,52]. The reaction mixture (200 μL), consisting of 2 mg/mL substrate, 50 mM Tris-HCl (pH 9.0), 0.5 M NaCl, and the purified AlyC7 (1.12 μg/mL), was incubated at 30 °C for 10 min. Reaction termination was achieved by boiling the mixture for 10 min. The increase in absorbance at 235 nm (A_235_) due to the release of unsaturated uronic in the mixture was monitored. One unit (U) of enzyme activity was defined as the amount of enzyme required to elevate A_235_ by 0.1 per minute. The optimum temperature for AlyC7 activity was investigated at pH 9.0 within a range of 20 to 50 °C. The optimum pH for AlyC7 activity was assayed at 30 °C in the Britton-Robinson (B-R) buffer, spanning pH 6.0 to 10.0 [31]. The effect of NaCl concentrations from 0.0 to 2.0 M on AlyC7 activity was determined at 30 °C and pH 9.0. The substrate specificity of AlyC7 was analyzed by measuring its activity towards PM, PG, PMG, and sodium alginate under the optimal reaction conditions.

The extracellular alginolytic activity of the recombinant *E. coli* strains was quantitatively determined by using the dinitrosalicylic acid (DNS) method [53,54]. Supernatants obtained by centrifuging the fermentation broths of the strains at 15,000× *g* at 4 °C for 5 min were used. The reaction mixture (200 μL) comprising 20 μL of supernatant, 2 mg/mL sodium alginate, 50 mM Tris-HCl (pH 9.0), and 0.5 M NaCl was incubated at 30 °C for 10 min. The reaction was halted by adding 100 μL of DNS, followed by boiling for 10 min. The absorbance value was determined at 540 nm, and the amount of reducing sugars released was quantified using glucose as the standard. One unit of enzyme activity was defined as the enzyme amount required to release 1 µg of reducing sugars from sodium alginate per minute.

### 3.6. Degradation Product Analysis

To analyze the degradation products of sodium alginate or alginate oligosaccharides by the purified recombinant AlyC7, the reaction mixture containing 2 mg/mL substrate, 1.2 mg/mL AlyC7, 50 mM Tris-HCl (pH 9.0), and 0.5 M NaCl was incubated at 30 °C for 3 h. The reaction was halted by adding 0.4 M trichloroacetic acid (TCA). Subsequently, the degradation products were subjected to high performance liquid chromatography (HPLC) analysis on a Superdex Peptide 10/300 GL column (GE Healthcare, USA) at a flow rate of 0.3 mL/min, with 0.2 M NH_4_HCO_3_ serving as the running buffer. The elution was monitored at 210 nm using a UV detector, and LabSolutions software facilitated real-time monitoring and data analysis. The degradation products of sodium alginate by the produced AlyC7 from strain PelB-AlyC7 were determined with the same method as described above.

The yield of trisaccharides from sodium alginate by the produced AlyC7 from strain PelB-AlyC7 was determined by HPLC on the aforementioned Superdex Peptide column with ultrapure water as the running buffer at a flow rate of 0.35 mL/min. A refractive index detector (RID) was employed for detection. A standard curve correlating trisaccharide concentration to peak area was constructed using a series of concentrations of the commercially available saturated mannuronate trisaccharide standard. The amount of trisaccharides released from sodium alginate by the produced AlyC7 was then calculated based on the standard curve.

### 3.7. Effects of Additives on AlyC7 Secretion by E. coli

Recombinant *E. coli* BL21 (DE3) strains were cultured in LB medium at 37 °C. Upon reaching an OD_600_ of 0.6, 0.3% (*w*/*v*) sorbitol, 0.3% (*w*/*v*) sucrose, 0.03% (*v*/*v*) SDS, 1% (*w*/*v*) glycine, or 1% (*v*/*v*) Triton X-100 was introduced to the cultures, and the strains were further cultured at 18 °C for 18 h in the presence of 0.3 mM IPTG. To analyze the effects of glycine or Triton X-100 concentrations on AlyC7 secretion, the culture broths of strains PelB-AlyC7, MalE-AlyC7, and OmpA-AlyC7 were supplemented with varying concentrations of these additives. The effects of these additives on AlyC7 secretion were assessed by measuring the extracellular alginolytic activity of the strains using the DNS method as described above.

### 3.8. Effects of Culture Conditions on AlyC7 Production by Strain pET22b-PelB-AlyC7

To determine the effect of IPTG concentration on AlyC7 production, strain PelB-AlyC7 was cultured in LB medium at 37 °C until the OD_600_ of the culture reached 0.6, IPTG was then added to the culture at concentrations ranging from 0 to 0.3 mM, and the culture was further incubated at 18 °C for 18 h. To determine the effects of induction temperature and time on AlyC7 production, strain PelB-AlyC7 was cultured in LB medium at 37 °C until the OD_600_ reached 0.6, and then 400 mM glycine was introduced to the culture. Subsequent incubation was carried out for varying durations at temperatures of 15, 18, 20, 22, or 25 °C. The effects of these culture conditions on AlyC7 production were assessed by measuring the extracellular alginolytic activity of strain PelB-AlyC7 using the DNS method.

### 3.9. Orthogonal Optimization

The optimal conditions for AlyC7 secretory production by strain PelB-AlyC7 were determined through an L_9_(3^4^) orthogonal experiment with three influencing factors, including induction temperature (factor A), glycine concentration (factor B), and induction time (factor C) (Table S4). Extracellular alginolytic activity was determined by using the DNS method.

### 3.10. Optimization of the Enzymolysis Parameters of the Produced AlyC7 on Sodium Alginate

The culture supernatant of strain PelB-AlyC7 was collected as the produced AlyC7. To determine the optimal E/S ratio and enzymolysis time, 2 mg sodium alginate was reacted with the produced AlyC7 at E/S ratios of 10, 25, 50, and 100 U/mg (enzyme activity determined by the DNS method), and the reactions were conducted in 50 mM Tris-HCl (pH 9.0) and 0.5 M NaCl at 30 °C, ranging from 10 to 100 min. Reactions were terminated by boiling the reaction mixtures for 10 min. The enzymolysis effect was evaluated by measuring the content of reducing sugars in the supernatant by the DNS method.

### 3.11. AOS Preparation by the Produced AlyC7

After enzymolysis of sodium alginate performed under optimal enzymolysis conditions by the produced AlyC7, ethanol was added to the reaction mixture at a final concentration of 70% (*v*/*v*), and the mixture was incubated at 4 °C for 12 h. After incubation, the mixture was centrifuged at 15,000× *g* at 4 °C for 5 min. The supernatant was then collected and freeze-dried to obtain AOS powder.

### 3.12. Effects of AOS on the Growth of Plant Seed Roots

The treatment of seeds was conducted following the methods previously reported, with minor modifications [55,56]. Seeds of wheat, maize, lettuce, and tomato were surface-sterilized with 0.05% (*v*/*v*) NaClO for 30 min and triple-rinsed with distilled water. Lettuce seeds were soaked on trays in distilled water or in solutions containing AOS (20, 50, 100, 200, or 300 µg/mL dissolved in distilled water) or sodium alginate (100 µg/mL dissolved in distilled water) in darkness at 18 °C for 3 days. Each group contained 100 seeds. After replacement with the same solutions, seedlings on trays were transferred to the light incubator with a day/night cycle of 16 h light/8 h dark at 28 °C. The solution was replaced on day 5, and root growth was observed on day 7. Tomato seeds were soaked on trays in distilled water or in solutions containing AOS (20, 50, 100, or 250 μg/mL) or sodium alginate (100 µg/mL) in darkness at 28 °C, with each group containing 30 seeds. The solution was replaced on day 2 and 4, and root growth was observed on day 5. Wheat seeds were soaked on trays in distilled water or in solutions containing AOS (5, 10, 20, 30, or 40 μg/mL) or sodium alginate (20 μg/mL) in darkness at 28 °C. Each group contained 100 seeds. The solution was replaced on day 2, and root growth was observed on day 4. Maize seeds were soaked on trays in distilled water or in solutions containing AOS (20, 150, 500, 750, or 1000 μg/mL) or sodium alginate (500 μg/mL) in darkness at 28 °C for 12 h. Each group contained 25 seeds. After soaking, seeds were planted in seedling-raising pots filled with perlite and vermiculite and placed in a light incubator with a day/night cycle of 16 h light/8 h dark at 28 °C. During the pot cultivation period, seeds were watered with distilled water every 24 h, and root growth was observed on day 7.

## 4. Conclusions

In this study, we characterized an alginate lyase, AlyC7, derived from a marine *Vibrio* strain, which was identified as a trisaccharide-producing lyase. Compared with previously reported trisaccharide-producing alginate lyases, AlyC7 exhibited broad substrate specificity, high enzymatic activity, and high trisaccharide production, suggesting its considerable potential for industrial production of alginate trisaccharides. To prompt its application, the extracellular production of AlyC7 in *E. coli* was explored by selecting signal peptides and additives, and culture conditions were optimized through single-factor and orthogonal experiments. Finally, a method for the extracellular production of AlyC7 in *E. coli* was successfully developed. This method, when compared with those reported for the extracellular production of other alginate lyases, offers advantages in terms of heightened extracellular alginolytic activity, reduced fermentation time, and a simplified medium and process. Furthermore, the enzymolysis conditions of the produced AlyC7 on sodium alginate were optimized. The resulting enzymolysis method enabled the rapid preparation of alginate trisaccharides, characterized by exceptional homogeneity and purity. The effects of the prepared AOS on plant root growth were assessed, revealing a significant promotion, particularly in the case of lettuce and tomatoes. The robust enzymatic performance of AlyC7, coupled with the efficiency of the extracellular production method and optimized enzymolysis conditions, underscores the potential of this study’s findings for advancing both fundamental understanding and practical applications in the realm of alginate trisaccharide production.

## Data Availability

The genome data of strain C42 has been submitted to the NCBI Genbank database under the accession number JAEKGD000000000.1. It can be found here: https://www.ncbi.nlm.nih.gov/nuccore/JAEKGD000000000.1/ (accessed on 21 December 2020). The amino acid sequence of AlyC7 has been submitted to the NCBI Genbank database under the accession number WP_010438370.1. It can be found here: https://www.ncbi.nlm.nih.gov/protein/WP_010438370.1 (accessed on 21 December 2020).

## Supplementary Materials

The following supporting information can be downloaded at: https://www.mdpi.com/article/10.3390/md22050230/s1, Table S1: Orthogonal experiment design and results of L_9_(3^4^); Table S2: Analysis of the results of orthogonal experiment; Table S3: The signal peptide sequences for the construction of the expression vectors; Table S4: Design of the orthogonal experiment.
