# Supplementary material for: High-Level Extracellular Production of a Trisaccharide-Producing Alginate Lyase AlyC7 in Escherichia coli and Its Agricultural Application
Source: Mar Drugs. 2024 May 18;22(5):230. doi: 10.3390/md22050230 (PMC11123115; doi:10.3390/md22050230)
Supplement: Supplementary file 1 [file marinedrugs-22-00230-s001.zip › marinedrugs-2981997-supplementary.pdf]

**Table S1.** Orthogonal experiment design and results of $L_9(3^4)$.

| Experiment number | Induction temperature (°C) (A) | Glycine concentration (mM) (B) | Induction time (h) (C) | Blank (D) | Extracellular alginolytic activity (U/mL) |
|---|---|---|---|---|---|
| 1 | 18 | 300 | 36 | 0 | 933.4 |
| 2 | 18 | 400 | 24 | 0 | 706.3 |
| 3 | 18 | 500 | 48 | 0 | 919.7 |
| 4 | 20 | 300 | 24 | 0 | 824.7 |
| 5 | 20 | 400 | 48 | 0 | 945.9 |
| 6 | 20 | 500 | 36 | 0 | 814.8 |
| 7 | 22 | 300 | 48 | 0 | 544.8 |
| 8 | 22 | 400 | 36 | 0 | 731.4 |
| 9 | 22 | 500 | 24 | 0 | 976.2 |

**Table S2.** Analysis of the results of orthogonal experiment.

| Parameters | | Level 1 | Level 2 | Level 3 |
|---|---|---|---|---|
| Extracellular alginolytic activity (U/mL) | $K_{Ax}$[a] | 853.2 | **861.8** | 750.8 |
| | $K_{Bx}$[a] | 767.6 | 794.5 | **903.6** |
| | $K_{Cx}$[a] | **835.8** | 826.5 | 803.5 |

[a] $K_{mx}$ represents the average targeting value of each factor and can be defined as $K_{mx}= G_{mx}/k_x$, where x (x=1, 2, 3) and m (m=A, B, C) indicate the level number and the factor, respectively. $G_{mx}$ denotes the sum of the targeting indexes of all levels in each factor m, and $k_x$ stands for the total level of the corresponding factor.

**Table S3.** The signal peptide sequences for the construction of the expression vectors.

| Signal peptide | Amino acid sequences | DNA sequences (5'-3') |
|---|---|---|

| | | |
|---|---|---|
| Natural signal | MKQITIKTLLASSILLAVG | ATGCGGGCGAAACTTCTGGGAATAGTCCTGACAACCCCTATTGCGATCAGCTCTTTT |
| PelB | MKYLLPTAAAGLLLLAAQPAMA | ATGAAATACCTGCTGCCGACCGCTGCTGCTGGTCTGCTGCTCCTCGCTGCCCAGCCGGCGATGGCC |
| MalE | MKIKTGARILALSALTTMMFSASALA | ATGAAAATAAAAACAGGTGCACGCATCCTCGCATTATCCGCATTAACGACGATGATGTTTTCCGCCTCGGCTCTCGCC |
| PhoA | MKQSTIALALLPLLFTPVTKA | ATGAAACAGTCGACTATTGCACTGGCACTGCTGCCGCTGCTGTTTACACCGGTAACAAAAGCA |
| OmpT | MRAKLLGIVLTTPIAISSFA | ATGCGGGCGAAACTTCTGGGAATAGTCCTGACAACCCCTATTGCGATCAGCTCTTTTGCT |
| OmpA | MKKTAIAIAVALAGFATVAQA | ATGAAAAAGACAGCTATCGCGATTGCAGTGGCACTGGCTGGTTTCGCTACCGTAGCGCAGGCC |

**Table S4.** Design of the orthogonal experiment.

| | Induction temperature (°C) (factor A) | Glycine concentration (mM) (factor B) | Induction time (h) (factor C) | Blank (factor D) |
|---|---|---|---|---|
| Level 1 | 18 | 300 | 24 | 0 |
| Level 2 | 20 | 400 | 36 | 0 |
| Level 3 | 22 | 500 | 48 | 0 |
